# Friends

**Published:** 1859-10

**Authors:** 


### "FRIENDS,"

Commonly called Quakers, are easily singled out from the throngers of Broadway by the dress, which, although the fashion of two hundred years ago, is no longer so, except to them. We never meet them without a respectful, affectionate drawing thither, accompanied with a sorrowful regret that there are not a hundred, where there is but one.

A true "friend" is the embodiment of plainness, placidity, and prosperity. The benevolent calmness which plays upon their features inspires in the beholder confidence and attachment.

But what is the secret of their universal power of presence and their general thrift? Who ever knew a Quaker to be in a fidget or a fix—the awful fix of having "nary red?" They neither beg nor steal, nor—we mean the "true blues"—cheat. The renegades, the half-and-half, the deserters, the impostors, and those who have been "hove overboard" neck and heels, we say nothing about. We are speaking of the stern and steady sort, who have never deserted their colors, and who have never been ashamed of their flag of drab.

The members of the Society of Friends have the kindly respect of all men—not the respect of fear, but that of love. Why thus? It is because of their distinguishing trait, that which is ever and all pervading, they act from a consciousness of justness. They wrong no man; they allow no man to wrong them. Or, if they do suffer wrong, it is for the sake of peace, but with a protest or a "testimony" against it, lest they might seem to connive at evil and wrong doing.

When a man acts always from a sense of justness, there is a freedom from fear, a confidence, a feeling of repose, which in time fixes on the whole character a calmness, a serenity, which is worth to its possessor more than gold. Hence there is a quiet in a Quaker, and a power too in that quiet, which would rout a regiment of fussy people in any contest. The heart, the conscience, the features, the very gait of a Friend, all are quiet—the glorious quiet, which nothing can ever give but an habitual consciousness of an all pervading rectitude of purpose. Thus it is that Friends do not fidget, and fret, and fritter their lives away, like the "world's people," as they call outsiders. English statistics show that their average age is some fifteen years more than that of others. The great secret, then, of a long and successful life is to "Do justly."

